# The role of rapamycin in the PINK1/Parkin signaling pathway in mitophagy in podocytes

**DOI:** 10.1515/biol-2022-0958

**Published:** 2024-09-09

**Authors:** Shengyou Yu, Weixue Zhu, Li Yu

**Affiliations:** Department of Pediatrics, Guangzhou First People’s Hospital, Guangzhou Medical University, Guangzhou, 510180, Guangdong Province, China; Department of Pediatrics, Guangzhou First People’s Hospital, School of Medicine, South China University of Technology, Guangzhou, Guangdong Province, P. R. China

**Keywords:** podocyte, rapamycin, VDAC1, mitophagy, signaling pathway

## Abstract

This study aimed to clarify the role of rapamycin in the PINK1/Parkin signaling pathway in mitophagy in podocytes and the role of voltage-dependent anion channel 1 (VDAC1) in the PINK1/Parkin signaling pathway in mouse glomerular podocytes. For this purpose, podocytes were cultured with rapamycin and observed using microscopy. The apoptosis rate of podocytes was detected by flow cytometry. Changes in the mitochondrial membrane potential were measured. The autophagy-related proteins VDAC1, PINK1, Parkin, and LC3 were detected, and mitochondrial autophagosomes were observed via transmission electron microscopy. In the present study, we demonstrated that the number of podocytes treated with rapamycin was significantly reduced. Compared with those in the control group, the apoptosis rate of podocytes and the degree of mitochondrial membrane potential depolarization were significantly higher. We also found the expression levels of VDAC1, PINK1, Parkin, and LC3 were significantly increased. In the rapamycin-treated group, the numbers of swollen mitochondria and mitochondrial autophagosomes were significantly higher. Finally, we showed that rapamycin can upregulate the expression of VDAC1, PINK1, Parkin, and LC3 in glomerular podocytes, which is correlated with mitophagy. VDAC1 is involved in mitophagy and is related to the PINK1/Parkin signaling pathway, serving as an indicator of mitophagy in podocytes.

## 1 Introduction

Inhibitors of the mammalian target of rapamycin (MTOR) belong to a family of drugs with potent immunosuppressive, antiangiogenic, and antiproliferative properties. Rapamycin was originally proposed as an immunosuppressant to prevent rejection of solid organ transplants. Despite its potential advantages in the transplant setting, evidence that sirolimus causes *de novo* or worsening proteinuria is unequivocal. In one study, disruption of the autophagic pathway may play a role in the pathogenesis of proteinuria in patients treated with MTOR inhibitors, but the mechanism by which this occurs is unknown [1]. The mTOR inhibitor rapamycin can reduce gene transcription and translation, leading to the activation of apoptosis signaling pathways. As a specific mTOR signaling pathway inhibitor, it can activate autophagy and mitophagy in damaged cells and mitochondria [2,3]. Mitophagy, the selective degradation of damaged and dysfunctional mitochondria via autophagy, is an important mechanism of mitochondrial quality control [4]. Mitophagy is the main pathway for the degradation of mitochondria [5,6]. It has been established that the specific mechanisms of mitophagy are primarily regulated by PINK1 (PTEN Induced Putative Kinase 1) and Parkin signaling pathway, and PINK1/Parkin-mediated mitophagy is one of the classical mitophagy pathways [7]. The PINK1/Parkin-mediated pathway involves protein phosphorylation and ubiquitination. VDAC1 is a mitochondrial outer membrane protein that forms a hydrophilic voltage-gated channel on the mitochondrial outer membrane that regulates the exchange of ions and small metabolites in and out of the mitochondria, forming a common pathway for exchanging metabolites between mitochondria and the cytoplasm. The common pathway plays a crucial role in the regulation of mitochondrial substances and energy functions [8]. VDAC1 is mainly involved in the ubiquitination process of the PINK1/Parkin signaling pathway and plays an integral role in mitophagy [9]. Therefore, rapamycin is essential for elucidating mTOR-dependent signaling events and their role in metabolism and disease; the use of this immunosuppressing drug for inhibiting the PINK1/Parkin signaling pathway pathway may serve as a new basis for the treatment of podocyte injury in renal disease. However, the effect of rapamycin on mitophagy in podocytes and VDAC1 is still unclear. In this study, we investigated the effect of rapamycin on normal podocytes, the effects of rapamycin on mitophagy in podocytes, and the effect of VDAC1 on the PINK1/Parkin signaling pathway in podocytes.

## 2 Materials and methods

### 2.1 Podocyte culture

*In vitro* immortalized mouse podocyte cell lines (MPC5) were authorized by the US Mundel Professor, Professor of Pediatrics, Peking University First Hospital, Ding Jie donation. Before the experiment, podocytes were recovered, collected, and cultured in RPMI 1640 medium (Gibco; Thermo Fisher Scientific, Inc.) containing antibiotics (1% penicillin and streptomycin) and 10% FBS (Gibco; Thermo Fisher Scientific, Inc.) at 33°C in a humidified 5% CO2/95% air atmosphere. Recombinant mouse γ-interferon (10 U/ml, BINDER GmbH) was used to induce podocyte proliferation. After podocyte confluence reached 80–90%, 0.25% trypsin/0.02% EDTA (Gibco; Thermo Fisher Scientific, Inc.) was added. Subsequently, the podocytes were moved to a 37°C environment for differentiation and maturation for 10–14 days. The differentiated and matured podocytes were then seeded into six-well plates (Corning), and after the cells reached 60–70% confluence, RPMI 1640 alone was used to starve the podocytes for 12 h to synchronize the proliferation of the cells, before the experimental grouping was performed.

Podocytes were treated with RPMI 1640 (Gibco, USA) supplemented with rapamycin (Med Chem Express, USA). Microscopy was used to observe the morphological changes in podocytes treated with different concentrations of rapamycin. The control group and the rapamycin group were designed as follows: control group: cells were treated with RPMI 1640 (Gibco, USA) nutrient solution containing 0.02% DMSO (Sigma, USA); rapamycin group: cells were treated with RPMI 1640 (Gibco, USA) nutrient solution containing 100 nmol/l rapamycin and 0.02% DMSO (Sigma, USA).

### 2.2 Flow cytometry analysis

The percentage of apoptotic podocytes was determined by Annexin V-FITC/propidium iodide (PI; Key GEN Biotech) dual staining. Changes in the mitochondrial membrane potential were detected with a JC-1 mitochondrial membrane potential detection kit (Key GEN Biotech). When the mitochondrial membrane potential is relatively normal, JC-1 gathers in the matrix of the mitochondria to form red-fluorescencent aggregates. When the mitochondrial membrane potential is decreased, JC-1 cannot accumulate in the matrix of the mitochondria and exists as green-fluorescencent monomer. JC-1 staining working solution was prepared according to the instructions. After 24 h, the culture medium was replaced by 1 mL fresh culture medium and 1 mL JC-1 staining working solution. The podocytes were further incubated at 37°C and JC-1 staining buffer was prepared according to the instructions during the incubation period. The JC-1 staining working solution was discarded, and the podocytes were washed twice with JC-1 staining buffer. After washing with phosphate-buffered saline (PBS) twice, the red fluorescent signals were excited to 530 nm and detected at 630 nm; the green fluorescence was excited to 488 nm and detected at 530 nm. Image J software was used to quantify the red and green fluorescence, and the ratios of red/green were calculated. Three independent experiments were carried out for validation.

### 2.3 RT-qPCR analysis

Total RNA was extracted according to the instructions of the RNA gents^®^ Total RNA Isolation System (Promega Corporation). Then, the purity and integrity of the total RNA was detected. Briefly, the purity of the RNA was detected by measuring the OD 260/OD 280 ratio with a nucleic acid protein analyzer (Bio Photometer Plus; Shanghai Musen Biotechnology Co., Ltd.). The integrity of the total RNA was assessed by 1% agarose gel electrophoresis, which was performed at 80 V for 20 min, and the total RNA bands were observed with a gel imaging system (Zhuhai Hema Medical Instrument Co., Ltd.). Thereafter, the extracted RNA was transcribed into cDNA using PrimeScript™ RT Master Mix Kit (Takara Bio, Inc.) according to the manufacturer’s protocol. SYBR Premix Ex Taq™ II (Takara Bio, Inc.) was used for qPCR, and reactions were performed using a LightCycler480 Real-Time PCR System (Roche Diagnostics) with the following protocol:predenaturation at 95°C for 5 min and denaturation at 95°C for 10 s and annealing at 60°C for 20 s for a total of 40 cycles. The VDACs1, PINK1, Parkin, and LC3 genes were quantitatively amplified with primers as described previously using the SYBR Green qPCR method [10,11,12,13]. The quantization of mRNA was calculated according to the attached software (Bio-Rad, USA) and normalized to the quality of β-actin.

### 2.4 Western blot analysis

The podocytes in each group were rinsed twice with PBS. RIPA cell lysate (Thermo, USA) was used for splitting decomposition. A BCA protein assay kit (Thermo, USA) was used to measure the total protein concentration of the podocytes. SDS–PAGE was performed in our laboratory according to previously described methods [14]. Afterward, the membranes were blocked with 5% (v/v) BSA for 1 h at room temperature. The primary antibodies for VDAC1, PINK1, Parkin, and LC3-B (Abcam, UK) were incubated at 4°C overnight. A Tanon 5200 (Tianneng Group) chemiluminescence imaging system was used for band exposure. The images were analyzed using ImageJ image analysis software.

### 2.5 Transmission electronic microscopy

Podocytes from each group were collected, fixed with glutaraldehyde and 1% osmium tetroxide for 15–30 min at 4°C, and dehydrated at room temperature with gradient concentrations of acetone. The dehydrated samples were embedded in pure acetone‑EPON812 embedding medium for 2 h at room temperature, baked to solidify, sectioned (1 µm), stained dropwise with sodium acetate for 30 min at room temperature, and washed with PBS. Samples were imaged using a transmission electron microscope (Libra 120 microscope; Carl Zeiss AG) to observe autophagosomes.

### 2.6 Statistical analysis

All the data were statistically analyzed with SPSS 20.0 statistical software (IBM Corp., Armonk, NY, USA). Count data are expressed as rates (%), and the *x* ^2^ test was used; measurement data are expressed as \[\bar{x}]\] ± S, and comparisons between groups were made using one-way analysis of variance. *p* < 0.05 indicated that the differences were statistically significant.

## 3 Results

### 3.1 Morphological changes of podocytes

**Figure 1:**
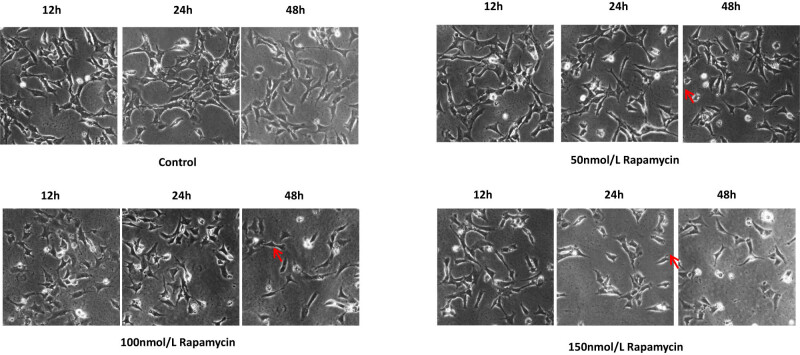
Changes in podocyte morphology in the control group and groups treated with different concentrations of rapamycin (inverted microscope ×200). The red arrows indicate the morphology of the podocytes.

Figure 1 shows that there was no obvious abnormality in the morphology of the podocytes treated with 50 nmol/l rapamycin compared with those in the control group. Podocytes treated with 100 and 150 nmol/l rapamycin showed a significant reduction in size, and the podocyte process retracted or disappeared. The connections among cells were loose. Consistent with other studies [11], the rapamycin group in the following experiment was constructed with a concentration of 100 nmol/l (Figure 1).

### 3.2 Changes in podocyte apoptosis rate

**Figure 2:**
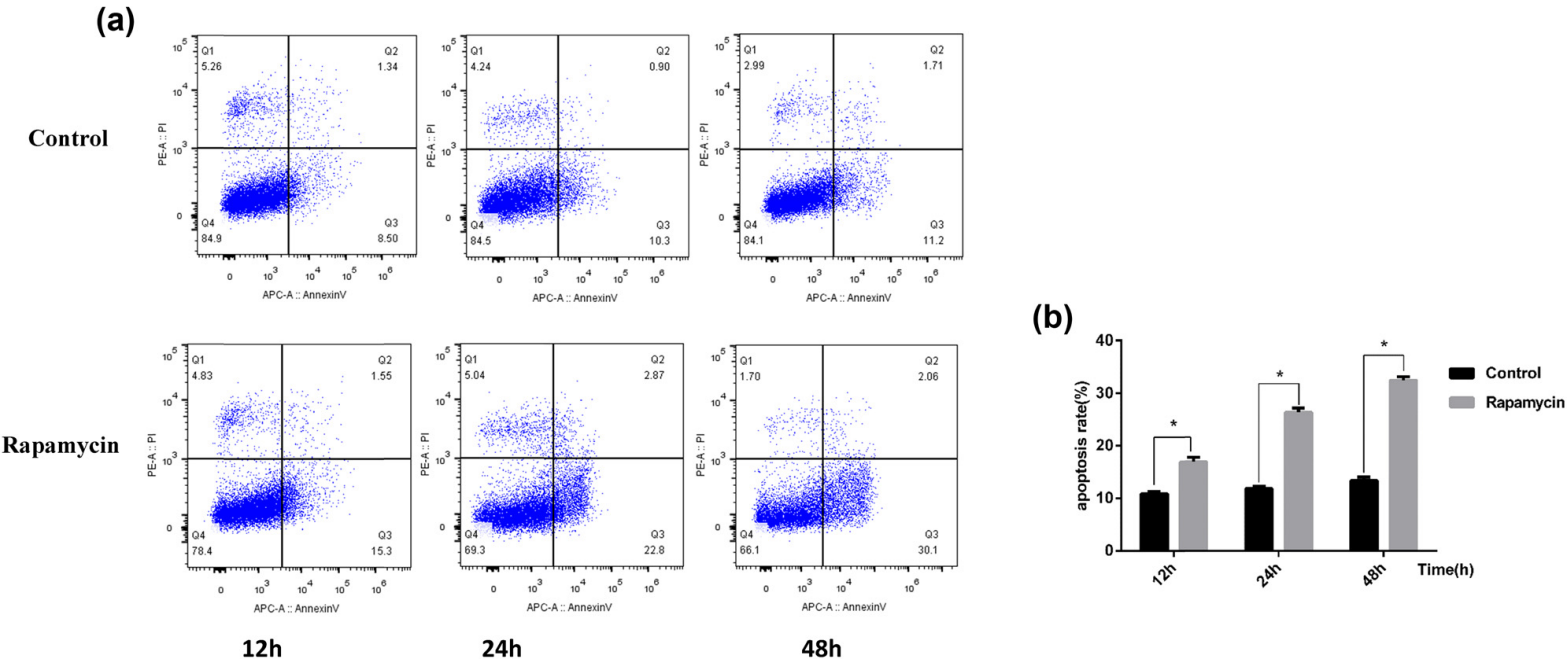
(a) The apoptosis rates of podocytes detected by flow cytometry in the control group and rapamycin group at different time points. Propidium iodide (PI) and an Annexin V apoptosis kit with double staining were used to detect the apoptosis rate, which was analyzed by flow cytometry, in which the *x*-axis was Annexin V(+) and the *y*-axis was PI (+), Q1 was PI (+), Annexin (–V), Q2 was PI (+), Annexin (+V), late apoptotic or dead cells, Q3 was PI (–), Annexin V (+), early apoptotic cells, Q4 was Annexin V (−), PI (–), and normal living cells. (b) Bar chart of the podocyte apoptosis rate detected by flow cytometry in the control group and rapamycin group. *p < 0.05 versus the control group.

In the control group, the apoptosis rate of podocytes at 12 and 24 h was low, and while the apoptosis rate slightly increased at 48 h, it was not significantly different (*p* > 0.05). After 12, 24, and 48 h of rapamycin treatment, the apoptosis rate of podocytes was significantly increased at 12 h compared to the control group (Figure 2).

### 3.3 Changes in podocyte mitochondrial membrane potential

**Figure 3:**
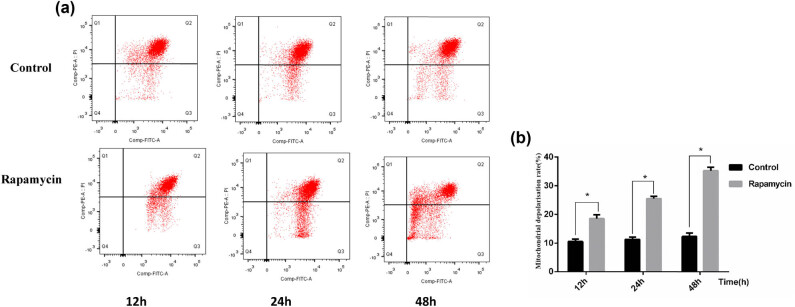
(a) Changes in mitochondrial membrane potential detected by flow cytometry in the control group and rapamycin group. (b) Bar chart of mitochondrial membrane potential detected by flow cytometry in the control group and rapamycin group. *p < 0.05 versus the control group.

In the control group, there were fewer mitochondrial JC-1 monomers at 12 h, and the number of monomers gradually increased with time, but the difference was not significant (*p* > 0.05). Compared with that in the control group, the monomer concentration in the rapamycin group increased at each time point, as shown in Figure 3. The number of monomers increased, indicating a greater degree of mitochondrial depolarization (*p* < 0.05; Figure 3).

### 3.4 mRNA expression levels of VDAC1, PINK1, Parkin, and LC3 in podocytes

**Figure 4:**
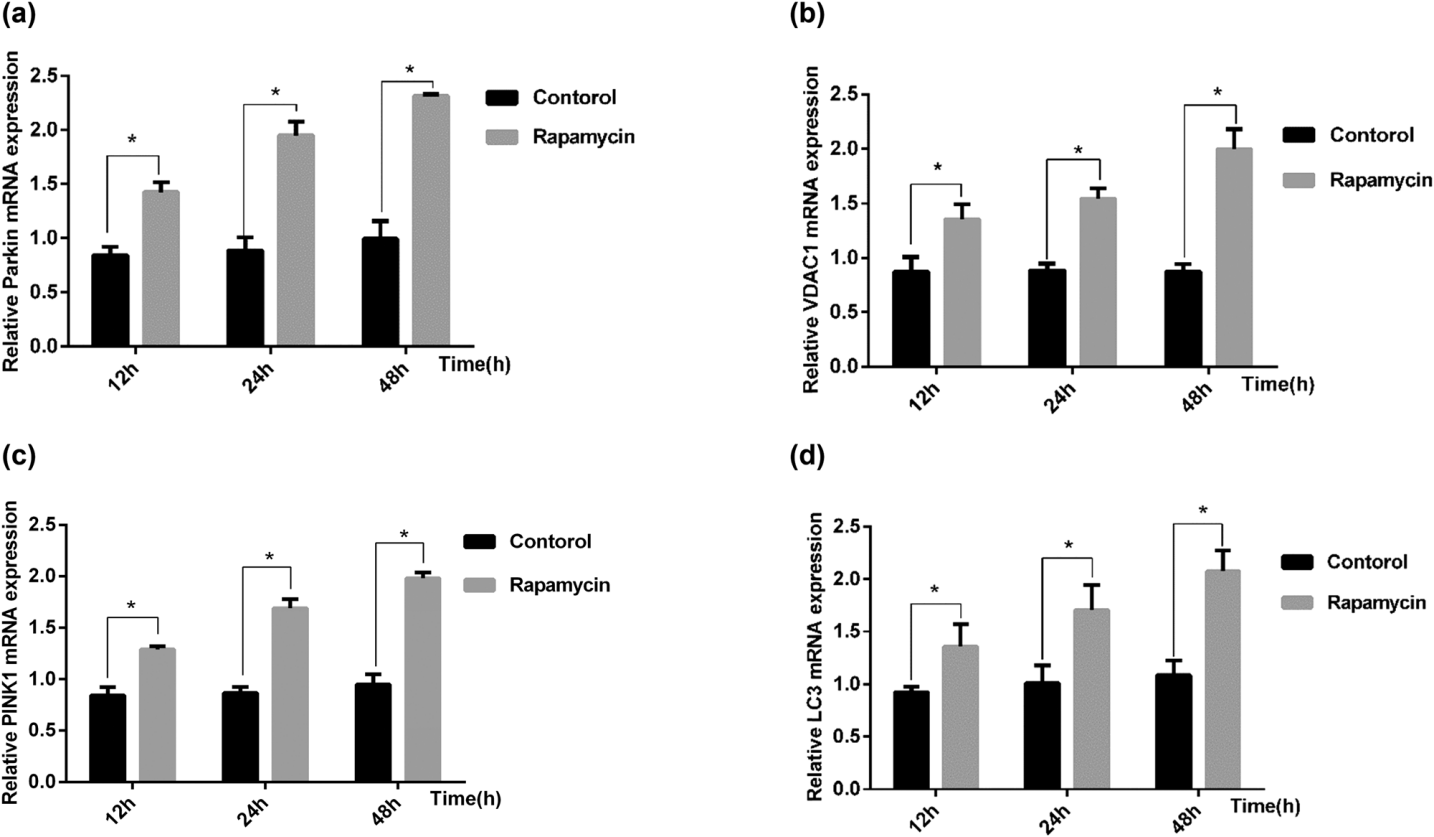
Changes in the mRNA expression of VDAC1, PINK1, Parkin, and LC3-B in the control group and rapamycin group. (a) VDAC1 mRNA expression, (b) the mRNA expression of PINK1, (c) the mRNA expression of Parkin, and (d) the mRNA expression of LC3-B. *p < 0.05 versus the control group.

In the control group, the mRNA expression of the mitophagy-associated genes VDAC1, PINK1, and Parkin and the autophagy-associated gene LC3 at 12 h was low, and the expression level did not change significantly with time (*p* > 0.05). Compared with those in the control group, the mRNA expression of VDAC1, PINK1, Parkin, and LC3-B in the rapamycin group significantly increased at each time point, and the expression gradually increased with time (*p* < 0.05; Figure 4).

### 3.5 Protein expression levels of VDAC1, PINK1, Parkin and LC3B in podocytes

**Figure 5:**
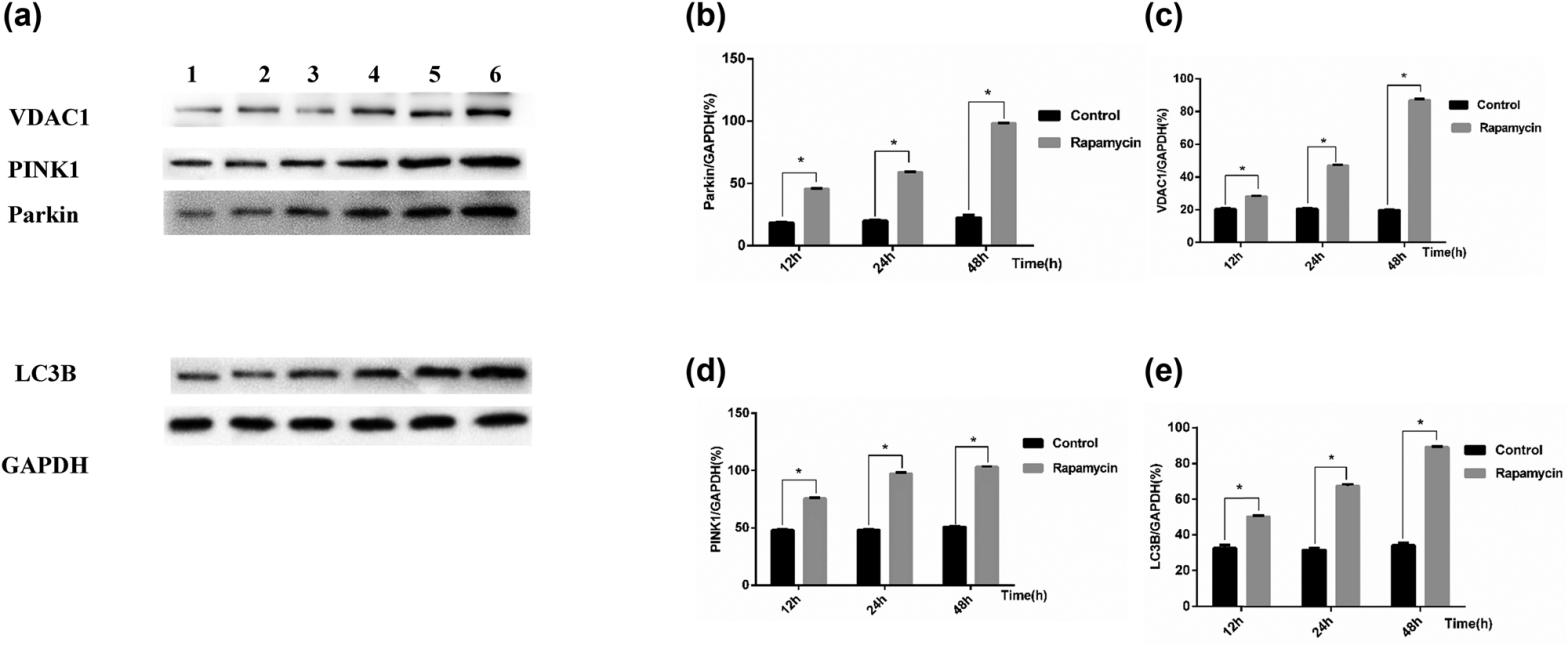
(a) Western blot bands of VDAC1, Pink1, Parkin, and LC3-B in the control group and rapamycin group at different time points. 1, 2, and 3 indicate the control group at 12, 24, and 48 h, respectively. 4, 5, and 6 indicate the rapamycin groups at 12, 24, and 48 h, respectively. Bar chart of the protein expression of VDAC1, PINK1, Parkin, and LC3-B in the control group and rapamycin group. (b) VDAC1 protein expression, (c) expression of the PINK1 protein, (d) Parkin protein expression, and (e) expression of the LC3-B protein. *p < 0.05 versus the control group.

In the control group, the expression of the mitophagy-related proteins VDAC1, PINK1, and Parkin and the autophagy-related protein LC3-B at 12 h was low, and their expression gradually increased with time, but there was no significant difference (*p* > 0.05). Compared with those in the control group, the expression levels of the proteins PINK1, Parkin, VDAC1, and LC3-B increased at each time point, and showed significant differences (*p* < 0.05; Figure 5).

### 3.6 Mitochondrial morphology and autophagosomes

**Figure 6:**
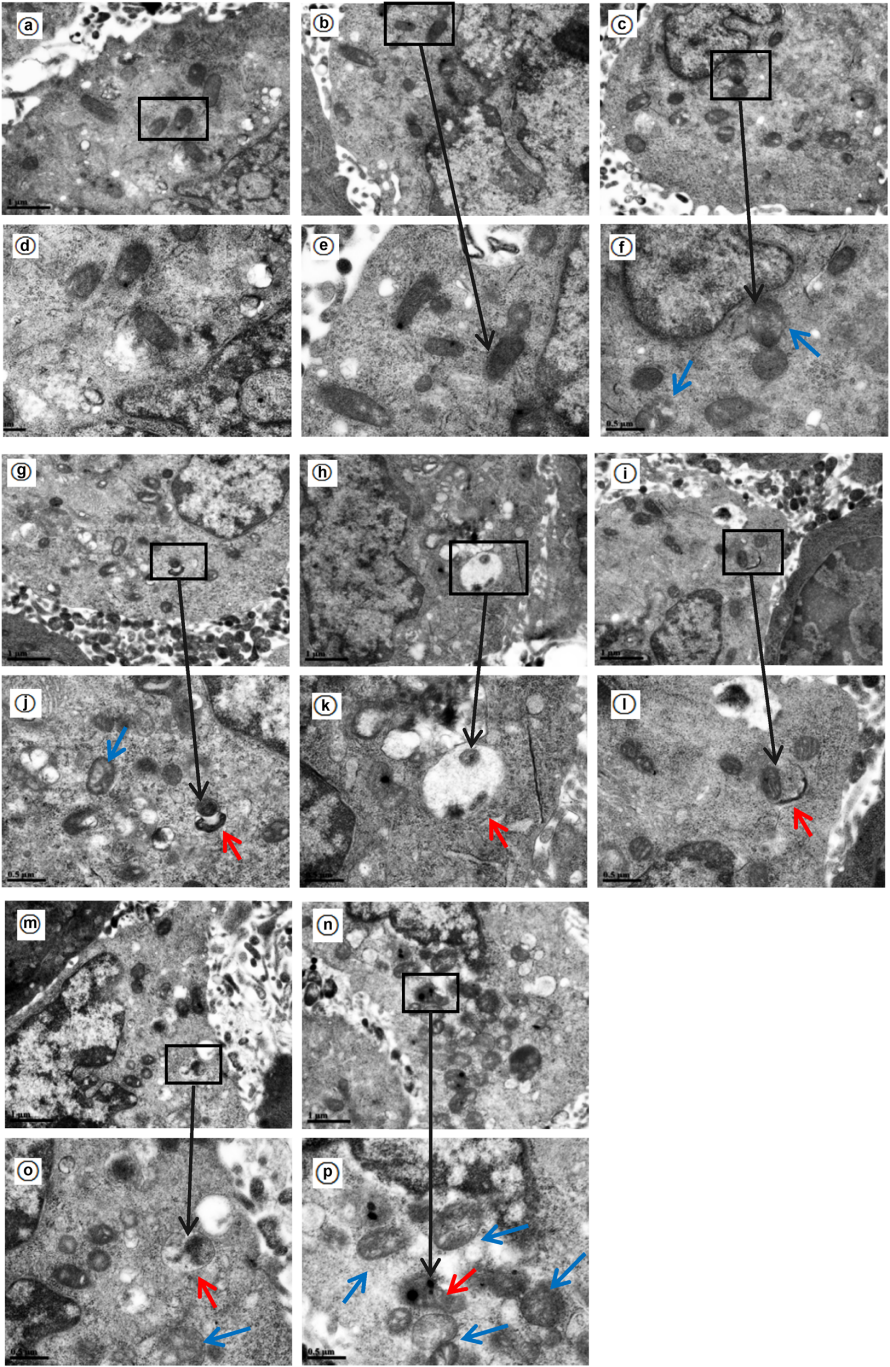
Changes in mitochondrial autophagosomes and mitochondria in the different groups were observed by transmission electron microscopy. (a)–(c) (×13,500), (d)–(f) (×26,500): the control group at 12, 24, and 48 h, respectively. (g) (×13,500), (j) (×26,500): rapamycin group at 12 h. (h)–(i) (X13500), (k)–(l) (×26,500): rapamycin group at 24 h. (m)–(n) (×13,500), (o)–(p) (×26,500): rapamycin group at 48 h. The blue arrows indicate swollen mitochondria, and the red arrows indicate autophagosomes.

In the control group, most of the mitochondria had clear structures with complete mitochondrial cristae. Several swollen mitochondria could be occasionally observed. No isolated bilayer-encapsulated mitochondrial autophagosomes were observed in multiple visual fields. At 48 h, the density of the podocytes was reduced, the volume was slightly reduced, and more lysosomal structures and swollen mitochondria were observed in the cytoplasm. In the rapamycin group, more swollen mitochondria were observed at various time points than in the control group, and as time progressed, the number of swollen mitochondria increased, some of them became vacuolated, and there were more lysosomes and lysosome-encapsulated autophagosomes (Figure 6).

## 4 Discussion

Mitochondria play a critical role in maintaining podocytic metabolic homeostasis. It has been demonstrated that Mitophagy is an important protective mechanism for eliminating damaged mitochondria and maintaining a healthy mitochondrial pool. Accumulating evidence has demonstrated that mitochondrial fragmentation is a prerequisite for the activation of mitophagy, which allows for the segregation of damaged mitochondria via the fission process and subsequent being engulfed by autophagosomes [15]. Mitophagy is a highly selective form of autophagy that eliminates dysfunctional or over-expressed mitochondria through the autophagosome-lysosomal system; this process is essentially giant autophagy [16].

At present, the most studied pathway for mediating mitochondrial autophagy is the PINK1/Parkin pathway, which is also the most recognized signaling pathway in mitophagy. Mitochondrial depolarization prevents the import of PINK1 to the inner membrane, leading to the accumulation of full-length PINK1 on the mitochondrial surface which recruits Parkin from the cytosol to damaged mitochondria. Following the recruitment to mitochondria, Parkin ubiquitinates various substrates to induce and promote the autophagic removal of mitochondria [17]. Under conditions of gene mutation, oxidative stress, or other factors, the mitochondrial permeability transition pore opens, and membrane potential (Δψ*m*) depolarization occurs [18]. PINK1 transport is blocked, and parkin is induced to move to the damaged mitochondrial outer membrane so that Parkin is phosphorylated and activated; subsequently, VDAC1 and other related proteins on the mitochondrial outer membrane are ubiquitinated, leading to the accumulation of the autophagy receptor p62. Then, damaged mitochondria are formed into autophagosomes through LC3 binding and enter lysosomes for degradation, eventually allowing the damaged mitochondria to be cleared by autophagy [19,20]. The link between PINK1, Parkin, and mitophagy has been studied in degenerative diseases, cardiovascular diseases, and tumors [21,22,23].

Voltage-dependent anion channels (VDACs) are also known as mitochondrial outer membrane proteins and play a crucial role in the regulation of mitochondrial substances and energy functions [24]. VDAC1 interacts with the bcl-xl family of antiapoptotic proteins, inhibits the release of apoptotic proteins, and participates in mitochondria-mediated apoptosis. Changes in the expression of VDAC1 affect cell material, energy metabolism, and cell survival; silencing VDAC1 expression by siRNA can reduce intracellular ATP levels and cell growth [25], while VDAC1 overexpression can induce cell death [26]. In addition, VDAC1 plays an integral role in PINK1/Parkin-mediated mitophagy [27,28,29]. It is a mitochondrial target of polyubiquitination and mitophagy mediated by Parkin, and VDAC1 is the target of lysine polyubiquitination mediated by Parkin during depolarization of mitochondria. Silencing the VDAC1 gene can significantly reduce Parkin migration from the cytoplasm to damaged mitochondria and significantly inhibit the elimination of mitochondria.

mTOR as a major negative regulator of mitophagy has been paid increasing attention. RAP is a macrolide immunosuppressive drug that can specifically bind to the mammalian target of rapamycin (mTOR) in the P13K/AKT/mTOR signaling pathway, inhibiting the protein kinase activity of mTOR, regulating cell metabolism, and inducing autophagy to regulate various cellular responses [30]. Under physiological conditions, the dynamic equilibrium of podocytes usually also relies on increased autophagy activity and very low mTOR activity. When the balance between mTOR activity and autophagy activity is disrupted, podocytes are damaged [31,32,33]. Additionally, rapamycin can cause the accumulation of PINK1 and the recruitment of Parkin to impaired mitochondria, followed by ubiquitination of the mitochondrial outer membrane protein VDAC1, activation of mitochondrial autophagy, and elimination of dysfunctional mitochondria [34]. Studies have shown that after cells are treated with rapamycin, the expression of cytochrome c and the activity of caspase-3 are greatly increased, so rapamycin can inhibit the PI3K/Akt signaling pathway and induce mitochondria-mediated apoptosis. In addition, abnormal mTOR activation in podocytes involved a variety of molecular reactions, including the mislocalization of slit diaphragm proteins in podocytes [35,36,37]. This was consistent with our study that the abnormal activation of mTOR played a key role in podocyte injury and proteinuria.

In the present study, we found that rapamycin can cause depolarization of the mitochondrial membrane potential of podocytes. The expression of VDAC1 increased, and the rate of apoptosis increased, which may be related to the fact that rapamycin can decrease the mitochondrial membrane potential. This led to the opening of mitochondrial permeability transition pores, promoted enhanced expression of cytochrome C, increased caspase-3 activity, and induced apoptosis of podocytes. In addition, rapamycin, an mTOR inhibitor, can significantly inhibit PI3K/Akt/mTOR signaling and activate caspase activity and cytochrome C release, further accelerating cell apoptosis [16,29]. In addition, we also found that rapamycin can increase the expression of mitophagy-associated proteins such as PINK1, Parkin, and VDAC1. Moreover, many swollen mitochondria, mitochondrial autophagosomes, and autolysosomes can be observed via transmission electron microscopy. We speculated that rapamycin caused podocyte mitochondrial dysfunction, increased Parkin migration from the cytoplasm to damaged mitochondria, enhanced VDAC1 expression, and promoted mitophagy in podocytes. Therefore, VDAC1 can participate in the process of podocyte mitophagy through the PINK1/Parkin signaling pathway. There is low mitophagy in normal cells. When rapamycin acts on podocytes, it can disrupt the original autophagy balance and lead to enhanced mitophagy, which leads to podocyte dysfunction and structural damage. When external stimuli are weak, mitophagy is activated to eliminate a small part of the damaged mitochondria, which protects cells, and excessive autophagy may cause the leakage of cathepsins or other proteolytic enzymes from lysosomes or autolysosomes, leading to cell apoptosis or necrosis.

## 5 Conclusion

Our findings indicate that enhancing autophagy and mitophagy can eliminate damaged organelles to a certain extent to maintain cell homeostasis, and overactivation may be detrimental to healthy organelles and disrupt cell homeostasis. Rapamycin can increase the protein expression of PINK1, Parkin, and VDAC1 by affecting the mitochondrial membrane potential, which promotes mitophagy mediated by the PINK1/Parkin signaling pathway. An imbalance in mitophagy in podocytes can result in increased cell apoptosis. VDAC1 is involved in mitophagy mediated by the PINK1/Parkin signaling pathway and can be used as an indicator to detect mitophagy in podocytes.
